# Estimating brain and eye lens dose for the cardiologist in interventional cardiology—are the dose levels of concern?

**DOI:** 10.1093/bjr/tqae089

**Published:** 2024-05-07

**Authors:** Markus Hulthén, Virginia Tsapaki, Angeliki Karambatsakidou

**Affiliations:** Department of Medical Radiation Physics and Nuclear Medicine, Karolinska University Hospital, 171 76 Stockholm, Sweden; Dosimetry and Medical Radiation Physics Section, Human Health Division, IAEA, Vienna, Austria; Department of Medical Radiation Physics and Nuclear Medicine, Karolinska University Hospital, 171 76 Stockholm, Sweden; Department of Oncology-Pathology, Karolinska Institutet, 171 76 Stockholm, Sweden

**Keywords:** conversion coefficients, brain dose, eye lens dose, radiation protection, radiation protection in interventional cardiology

## Abstract

**Objectives:**

To establish conversion coefficients (CCs), between mean absorbed dose to the brain and eye lens of the cardiologist and the air kerma-area product, *P*_KA_, for a set of projections in cardiac interventional procedures. Furthermore, by taking clinical data into account, a method to estimate the doses per procedure, or annual dose, is presented.

**Methods:**

Thermoluminescence dosimeters were used together with anthropomorphic phantoms, simulating a cardiologist performing an interventional cardiac procedure, to estimate the CCs for the brain and eye lens dose for nine standard projections, and change in patient size and x-ray spectrum. Additionally, a single CC has been estimated, accounting for each projections fraction of use in the clinic and associated *P*_KA_ using clinical data from the dose monitoring system in our hospital.

**Results:**

The maximum CCs for the eye lens and segment of the brain, is 5.47 μGy/Gycm^2^ (left eye lens) and 1.71 μGy/Gycm^2^ (left brain segment). The corresponding weighted CCs: are 3.39 μGy/Gycm^2^ and 0.89 μGy/Gycm^2^, respectively.

**Conclusions:**

Conversion coefficients have been established under actual scatter conditions, showing higher doses on the left side of the operator. Using modern interventional x-ray equipment, interventional cardiac procedures will not cause high radiation dose levels to the operator when a ceiling mounted shield is used, otherwise there is a risk that the threshold dose values for cataract will be reached.

**Advance in knowledge:**

In addition to the CCs for the different projections, methods for deriving a single CC per cardiac interventional procedure and dose per year were introduced.

## Introduction

Interventional cardiology (IC) procedures have become routine across the world as they are an efficient alternative to open surgery, extracorporeal circulation or lengthy stay within the hospital and they are the main tool for a successful clinical outcome. The use of x-ray imaging technology plays a pivotal role in this process and the evolution of technology boosted its use shifting steadily from analogue to digital detectors from simple fluoroscopy machines to complex sophisticated angiography systems with outstanding processing tools to address many different clinical problems.^1–3^ The wide accessibility of modern x-ray devices together with increased patient demand has resulted in their use outside the traditional radiology department by other clinical specialists such as interventional cardiologists.

The use of ionising radiation in medical imaging and more specifically in angiography systems is, however, associated with various risks including the risk of cancer or immediate effects and they should be optimised.^4^^,^^5^ Taking the great benefits of IC procedures for granted the reports of patient radiation dose studies have raised concern regarding radiation dose levels.^6–8^ IC procedures are thus defined as high-dose procedures,^9^ exposing high radiation doses to patients and thus to the medical staff as well.

Although the clinical benefit to the patient has been shown to be much greater than the radiation risks, the corresponding occupational radiation risks are not always properly considered. According to recent literature,^5^ focus the last years has been on the new International Commission on Radiological Protection (ICRP) recommended limit on the equivalent dose for the lens of the eye; 20 mSv in a single year, or 100 mSv in five consecutive years subject to a maximum dose of 50 mSv in a single year. This was proposed by ICRP in Publication 118,^10^ advanced in the ICRP Statement on Tissue Reactions and later adopted by the International and European BSS.^11^ There are publications in the recent literature that focus on radiation related operator risks such as risk for lens opacity^12–16^ and brain tumours.^17–19^ It must be noted that no epidemiological studies have proven the incidence of cataract^20^ or brain tumours^21^^,^^22^ caused by radiation for interventional cardiologists. On the other hand, significant correlation has been shown between incidence of cataract/brain tumours and radiation for higher radiation doses.^23^^,^^24^ Interestingly enough the brain tumours in most cases have been reported on the left side of the brain, that is, the operator side, that is closest to the tube and therefore is most exposed to the scattered radiation. Few studies have published equivalent organ dose to the operator’s eye lens^25–28^ and conversion coefficients (CCs), in terms of absorbed organ dose per unit patient air kerma area product (*P*_KA_), as reported by the x-ray system, to the operator’s eye lens^29–31^ and brain,^32^ as well. Other studies derived CC: s for estimating the dose to the brain normalised to dose at left collar level,^33^ and the personal dose equivalent [*H*_p_(10)]^34^ rather than *P*_KA_.

The contradictory findings,^17–19^^,^^21^^,^^22^ on whether brain tumours among interventional cardiologists can be attributed to occupational radiation exposure or not, was the driving force of the current study. The main objective was to develop a dose monitoring tool for estimating eye lens and brain doses to operators performing IC procedures. The tool consists of CCs, in terms of absorbed organ dose to the operator’s eye lens and brain, per unit *P*_KA_, obtained from phantom measurements, simulating an IC procedure using femoral puncture. As previous studies have shown,^31^^,^^32^^,^^34^ the CCs can greatly vary depending on the irradiation projection used. Therefore, in this work, CC: s calculated from a range of projections are weighted with clinical exposure data, enabling single CCs per procedure, for the brain and eye lenses, to be derived in any clinic. Normalisation to *P*_KA_ was chosen to exclude no users in IC, as *P*_KA_ is mandatory dose indicator,^35^ but personal dosimeters are not.

## Methods

### X-ray equipment

All measurements were performed with a monoplane Philips Allura Clarity X-ray angiography system (Philips, Best, The Netherlands). The system has a flat panel detector (FPD) with the three selectable Field-of-Views 15, 20, and 25 cm and an antiscatter grid (ratio, 13:1). Source to isocentre distance is 76.5 cm and, consequently, source to Patient Entrance Reference Point (PERP) distance 61.5 cm. A calculated *P*_KA_-value is obtained from the X-ray unit, which is checked against a reference instrument (Diamentor; PTW-Freiburg, Germany) calibrated for incident *P*_KA_-value at the Swedish secondary standard dosimetry laboratory. For this check the tandem model^36^ was used, with the only difference being the distance between the collimator and the reference *P*_KA_-meter, which was 15 cm instead of the recommended 30 cm. The deviation from the reference *P*_KA_ meter is <5%. Furthermore, the system uses automatic dose rate control (ADRC), which adjusts beam quality depending on patient size and type of examination.

### Phantom measurements—setup

**Figure 1.**
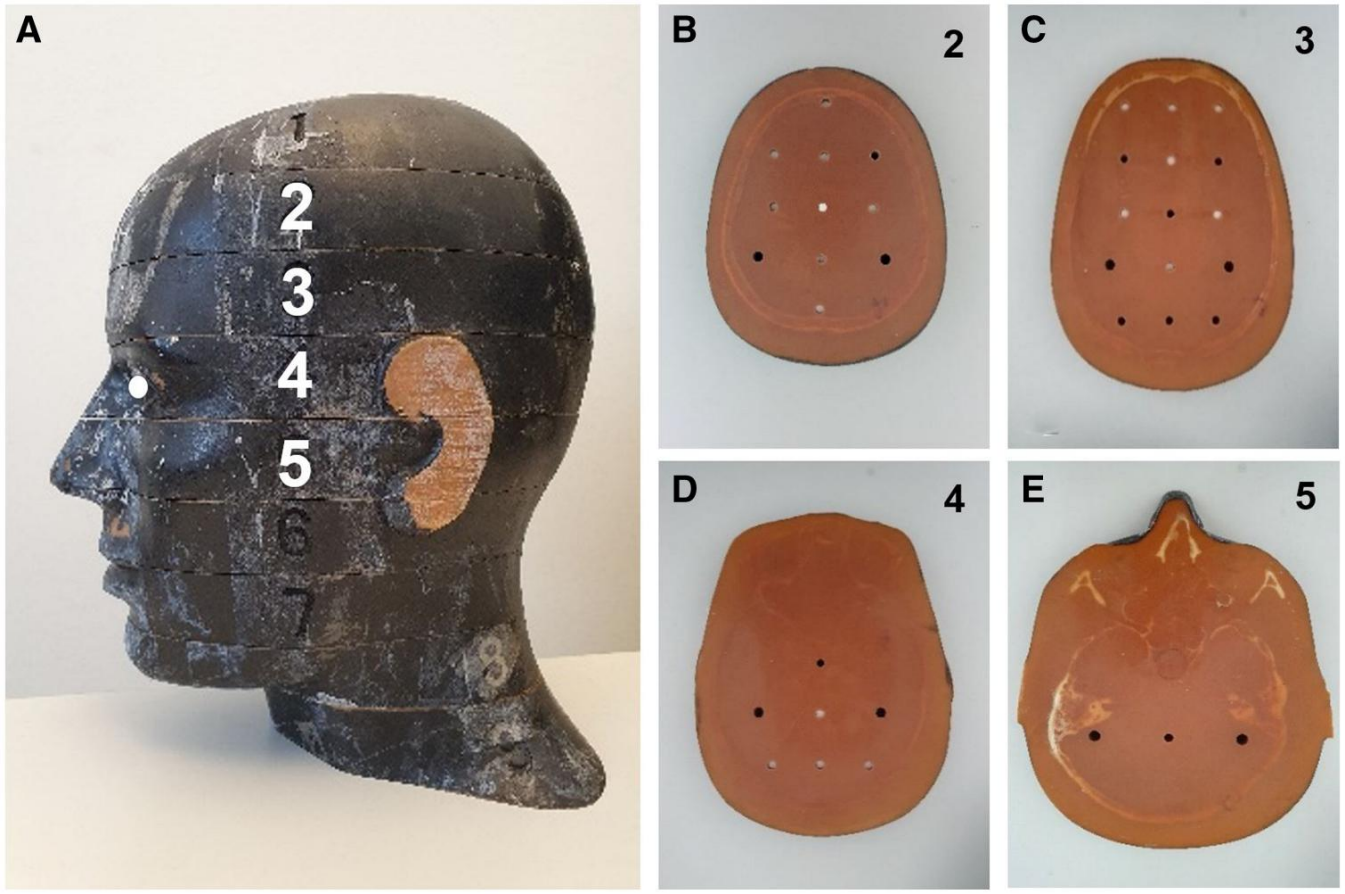
(A) The head of the Alderson-Rando phantom with the numbering of the slices marked. (B-E) the different slices (2-5) with the drilled holes for the position of the TLDs inside the brain.

The Multipurpose Chest Phantom N1 “Lungman” (Kyoto Kagaku Co, Japan) was used to mimic a patient undergoing a cardiological interventional procedure, hence serving as a scattering object. The phantom measures 20 cm in the anterior-posterior (AP) direction and approximately 30-43 cm in the lateral direction (30-35 cm at heart level). Chest circumference is 94 cm. It can be equipped with two additional soft tissue plates (each 30 mm thick) at the front and back in order to simulate a thicker patient.^37^ The Alderson-RANDO head Phantom (Radiology Support Devices, Long Beach, CA, United States) was used to mimic the main cardiologist performing the procedure. The phantom (Figure 1A) is horizontally transected in 2.5 cm thick slices and has a grid pattern of drilled holes inside the brain where Thermoluminescent Dosimeters (TLDs) can be inserted (Figure 1B-E). Dosimeters of type TLD-700 (LiF: Mg; Ti) were used. The TLDs were calibrated in terms of absorbed dose to water using a Harshaw Sr-90/Y-90 Irradiator 2000 and read out using a Harshaw QS 5500 reader (Thermo Fisher Scientific, Waltham, MA, United States). The smallest detectable dose is 10 µGy^38^ and the standard uncertainty from the TLD readings is ±2% and traceable to the national standard dosimetry laboratory. In slice no. 2-5 of the head phantom (Figure 1B-E), two TLDs were placed in each of the drilled holes. Furthermore, two TLDs were placed on the phantom’s left eye and right eye, respectively, marked with a white dot in Figure 1A.

**Figure 2.**
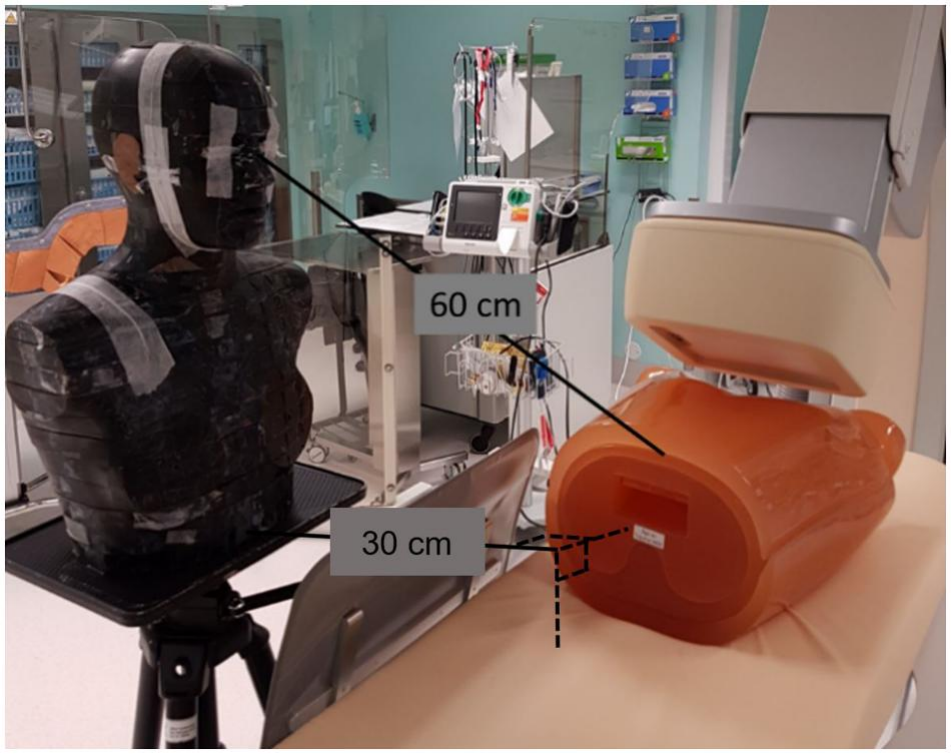
Experimental set-up of the Kyoto Kagaku N1 (Lungman) chest phantom with soft tissue plates on the patient table during the cranial 30° measurement. The Alderson-Rando phantom is placed in the position of an interventional cardiologist performing a procedure using femoral artery puncture. The height of the operator is 172 cm.

The chest phantom with the extra chest plates was placed on the patient table in a supine position and the head phantom was positioned on a tripod. The tripod was adjusted so that the top of the phantom was 172 cm above the floor, which is the median height of the Swedish population. With assistance from an interventional cardiologist in our hospital, the tripod was positioned so that the location of the phantom coincided with that of the main operator performing an interventional cardiac procedure utilising femoral artery access. The c-arm of the x-ray equipment and patient table were positioned in accordance with clinical routine, that is, with the heart of the chest phantom at isocentre, the FPD close to the patient (a few cm gap at its nearest location), and the X-ray field collimated to the area of interest, that is, the heart (Figure 2). Table height was 92 cm above the floor. This resulted in source to image receptor distances (SID) in the range of 100-113 cm (Table 3) and a beam size at the entrance surface of the FPD of 11 × 11 cm. No magnification was used.

**Table 1.** List of equipment settings for each measurement with respect to technique, tube potential, total filtration, rotation and angulation of the c-arm.

Projection name	Tube potential (kVp)	Technique	SID (cm)	Total filtration	Rotation	Angulation
PA	69	Digital image acquisition	100	4.5 mm Al + 0.1 mm Cu	0°	0°
LAO30	74	Digital image acquisition	103	4.5 mm Al + 0.1 mm Cu	30° LAO	0°
RAO30	68	Digital image acquisition	102	4.5 mm Al + 0.1 mm Cu	30° RAO	0°
CAUD30	70	Digital image acquisition	108	4.5 mm Al + 0.1 mm Cu	0°	30° CAUD
CRAN30	71	Digital image acquisition	103	4.5 mm Al + 0.1 mm Cu	0°	30° CRAN
LAO30/CAUD30	71	Digital image acquisition	108	4.5 mm Al + 0.1 mm Cu	30° LAO	30° CAUD
LAO30/CRAN30	71	Digital image acquisition	106	4.5 mm Al + 0.1 mm Cu	30° LAO	30° CRAN
RAO30/CAUD30	72	Digital image acquisition	113	4.5 mm Al + 0.1 mm Cu	30° RAO	30° CAUD
RAO30/CRAN30	70	Digital image acquisition	112	4.5 mm Al + 0.1 mm Cu	30° RAO	30° CRAN
LAO30_XL	89	Digital image acquisition	107	4.5 mm Al + 0.1 mm Cu	30° LAO	0°
LAO30_FL	88	Fluoroscopy	102	4.5 mm Al + 0.4 mm Cu	30° LAO	0°

Abbreviations: CAUD = caudal; CRAN = cranial; FL = fluoroscopic irradiation technique; LAO = left anterior oblique; PA = posterior-anterior projection; RAO = right anterior oblique; SID = source to image receptor distance; XL = extra-large patient.

**Table 2.** Attributes for dividing the brain into regions based on phantom slice number and pixel number in the acquired CT images of the phantom.

Region	Slice no.	Pixel no. (*x*)	Pixel no. (*y*)
Upper	2, 3	–	–
Lower	4, 5	–	–
Left	–	≥248	–
Right	–	≤247	–
Front	–	–	≤262
Rear	–	–	≥263

Only pixels inside the brain with the corresponding attributes belong to a region.

**Table 3.** Calculated CCs (absorbed dose to water per kerma area product) in units of µGy/Gy cm^2^ from each measurement to different regions of the brain.

	Whole brain	Left brain	Right brain	Upper brain	Lower brain	Front brain	Rear brain	Left eye	Right eye
PA	0.36	0.47	0.25	0.30	0.47	0.45	0.32	1.69	1.09
LAO30	0.92	1.18	0.66	0.88	0.99	1.07	0.83	3.87	1.78
RAO30	0.34	0.40	0.27	0.34	0.34	0.44	0.28	1.76	1.52
CAUD30	0.32	0.39	0.25	0.35	0.26	0.41	0.27	1.17	0.80
CRAN30	0.80	0.99	0.60	0.74	0.91	1.01	0.68	4.78	3.69
LAO30/CAUD30	0.63	0.84	0.42	0.54	0.79	0.66	0.62	2.03	0.91
LAO30/CRAN30	0.82	1.00	0.63	0.77	0.90	1.05	0.68	5.47	4.83
RAO30/CAUD30	0.14	0.15	0.12	0.12	0.17	0.15	0.12	0.52	0.46
RAO30/CRAN30	0.77	0.92	0.62	0.79	0.74	1.09	0.59	4.40	4.01
LAO30_XL	0.85	1.07	0.62	0.80	0.94	1.00	0.76	3.56	1.66
LAO30_FL	1.33	1.71	0.94	1.30	1.39	1.59	1.18	5.04	2.56
WEIGHTED	0.70	0.89	0.52	0.66	0.79	0.86	0.61	3.39	2.32

Abbreviations: CAUD = caudal; CRAN = cranial; FL = fluoroscopic irradiation technique; LAO = left anterior oblique; PA = posterior–anterior projection; RAO = right anterior oblique; XL = extra-large patient; WEIGHTED, based on each projection with their corresponding fraction of use including digital image acquisition and fluoroscopy mode.

The regions are divided as described in Table 2.

### Phantom measurements—beam geometry

For the first measurement, the c-arm was positioned in a posterior-anterior (PA) projection. In subsequent measurements, the c-arm was rotated and/or angulated 30° in left anterior oblique (LAO), right-anterior oblique (RAO), cranial (CRAN) and caudal (CAUD) directions and their possible combinations, respectively. Irradiation was performed using digital image acquisition mode (0.07 μGy/frame at the entrance surface of the FPD, 7.5 frames/s), rather than fluoroscopy in order to achieve dose rates high enough in the measurement positions and hence avoid impractically long irradiation times. For the same reason no ceiling suspended radiation shield was used but only the lower body protection positioned in front of the cardiologist. The *P*_KA_ ranges for the different projections were 300-500 Gy cm^2^. Settings for each measurement are listed in Table 1.

### Phantom measurements—beam quality and patient size

Two additional measurements were performed; one using fluoroscopy (0.19 μGy/s at the entrance surface of the FPD, 7.5 pulses/s) to investigate the effect of beam quality, and one using extra bolus material (Superflab Bolus, 5 cm thick) on the front of the chest phantom to simulate a thicker patient. Previous studies^31^^,^^39^^,^^40^ suggest that the operator is subjected to more scatter from LAO projections compared to PA or RAO. Therefore, the 30° LAO projection was used to increase dose rate and improve statistics in the measurements. *P*_KA_ was larger than 300  and 400 Gy cm^2^ for the two measurements, respectively, and the fluoroscopy time for the fluoroscopy measurement was approximately 10 h. All settings are listed in Table 1.

### Dose CCs

The absorbed dose to water (*D*_w_) was obtained by multiplying the reading from each TLD with an individual calibration factor and energy correction factor. The mean value of the doses from the two TLD dosimeters in each measurement position was taken to be the actual dose in that position. By dividing the calculated *D*_w_ with the total air kerma-area product (*P*_KA_) from a measurement, the CC (*D*_w_/*P*_KA_)^41^ in each position for all measurements was obtained.

For every measurement, two-dimensional (2D) distribution maps of the CCs in each slice of the head phantom containing TLDs were created. CT images of the head phantom were acquired, and the calculated values of the CCs were assigned to their corresponding positions in the axial image reconstructions. The distribution maps were created using 2D interpolation (radial basis functions from the Python SciPy library) between the measurement positions. Based on phantom slice number and pixel number in the CT images, the brain was divided into regions (Table 2) and the mean CC of the pixels in the distribution maps for each region was calculated.

### Weighted dose CC

The scatter to the operator is expected to depend on projection and beam quality. To provide a single more useful CC, the actual clinical distribution of *P*_KA_ in terms of rotation and angulation must be considered. Data from two catheterisation laboratories for two consecutive months were exported from our hospital’s dose monitoring system (Sectra Dosetrack, Sectra AB, Sweden), resulting in a dataset of 25 552 unique irradiation events from 249 cardiac procedures. The events were placed in bins ranging from −45° to +45° rotation and/or angulation in steps of 30 °, resulting in nine bins, each corresponding to one of the nine standard projections in this study. Binning was performed for both fluoroscopy and digital image acquisition. Since only one measurement was conducted using fluoroscopy (using LAO30 projection), the corresponding CCs using fluoroscopy for other projections were calculated by multiplying the CC from each projection’s digital image acquisition measurement, with the ratio between the CC for fluoroscopy and digital image acquisition using LAO30. Finally, the sum of the *P*_KA_ in each bin was divided with the total *P*_KA_ to obtain the fraction in that bin. By weighting the CC from each projection with their corresponding fraction of use, a weighted CC was calculated representing the actual clinical exposure to the operator.

## Results

All calculated CCs for eye lenses and the respective regions of the brain are presented in Table 3. The CCs are up to a factor of 2.2 and 2.0 higher on left side compared to the right side for the eye lenses and brain, respectively. Using the weighted CCs, the difference becomes a factor of 1.5 and 1.7 higher on the left side. TLD readings were in range of 0.03-2.40 mGy, well above the smallest detectable value (10 µGy).

**Figure 3.**
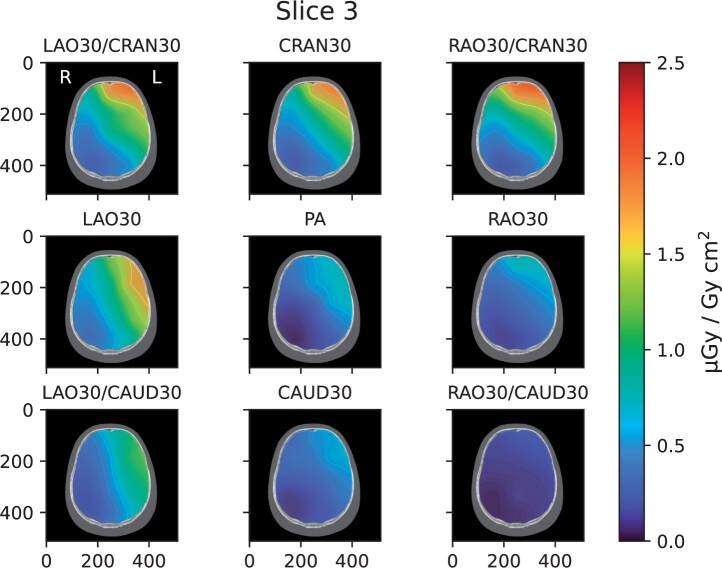
Distribution maps of the CC [absorbed dose to water (*D*_w_) in the brain per unit air kerma area product (*P*_KA_)] in slice no. 3 of the head phantom for each projection using digital image acquisition. Note that the orientation of the images is that of standard axial CT images, that is, they are viewed from the bottom of the phantom towards the top.

**Figure 4.**
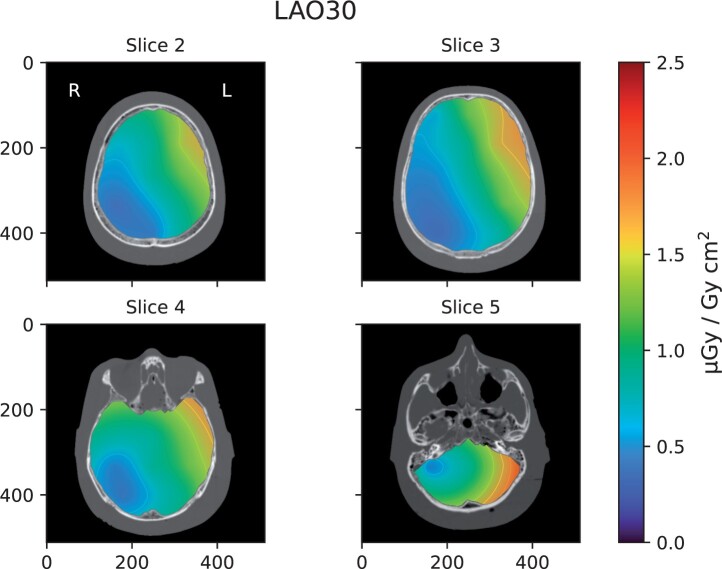
Distribution maps of the CC [absorbed dose to water (*D*_w_) in the brain per unit air kerma area product (*P*_KA_)] for each slice of the head phantom in the LAO 30° projection using digital image acquisition. Note that the orientation of the images is that of standard axial CT images, that is, images are viewed from the bottom of the phantom towards the top.

**Figure 5.**
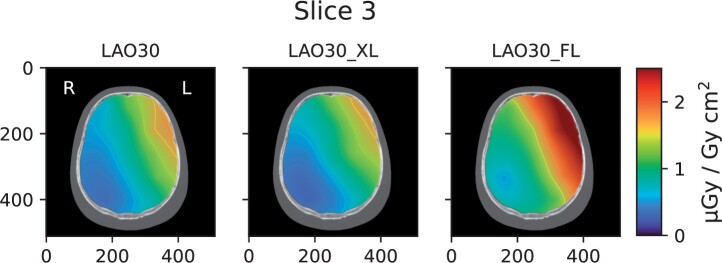
Distribution maps of the CC [absorbed dose to water (*D*_w_) in the brain per unit air kerma area product (*P*_KA_)] in slice no. 3 of the head phantom in the LAO 30° projection using digital image acquisition (left panel), digital image acquisition with a larger patient (centre panel) and fluoroscopy (right panel), respectively. Note that the orientation of the images is that of standard axial CT images, that is, they are viewed from the bottom of the phantom towards the top.

**Figure 6.**
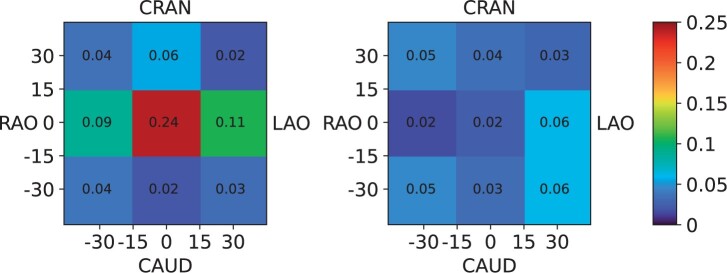
Fraction of total air kerma area product (*P*_KA_) originating from fluoroscopy (left panel) and digital image acquisition (right panel), respectively, for irradiation angles from −45 to 45° rotation and/or angulation in 30° bins. Data from 24913 irradiation events from 249 cardiac procedures performed during two consecutive months in our hospital. Note that the data does not necessarily reflect the clinical distributions in other hospitals.

Figures 3-5 show the dose distribution from scattered radiation per unit *P*_KA_ inside the brain of the operator, from each measurement. In Figure 3, the distribution maps in slice no. 3 of the head phantom for all standard projections using digital image acquisition are shown. To conserve space, distribution maps for the remaining slices (Figure 4) are shown for LAO30 only, and for the measurements with differences in patient size and beam quality (Figure 5) for slice no. 3 only. Figure 6 shows the distribution of *P_KA_* in terms of rotation and/or angulation for the two catheterisation laboratories for fluoroscopy and digital image acquisition, respectively. Out of the 25 552 irradiation events >97.5% (*n* = 24 913) were in the range of −45° to +45° rotation and/or angulation. Total *P*_KA_ in this interval was 5324.6 Gy cm^2^ out of which 3415.5 Gy cm^2^ (64.1%) originated from fluoroscopy. The PA- and LAO-projections in fluoroscopy, and LAO-CAUD-projections in digital image acquisition, yield the highest fractions of total *P*_KA_.

## Discussion

The dose measurements in this study provide a detailed mapping of the dose distribution inside the cardiologist’s brain, owing to scattered radiation from interventional cardiac procedures. Results show that the absorbed dose to the brain in interventional cardiologists performing procedures using femoral artery access are higher in the left side compared to the right, in the lower part compared to the upper and in the front part compared to the rear. A substantial difference between the left- and the right segment of the brain was found in the current study, being up to a factor of two higher in the left segment. Ferrari et al^32^ estimated CCs for the brain dose to the first and second operator in interventional radiology procedures using Monte Carlo simulations. The position of the second operator relative to the x-ray equipment was close to that of the operator in our study, resulting in a similar difference in dose to the left and right side of the brain, respectively. Fetterly et al^33^ irradiated a Rando phantom using scatter equivalent x-ray beams and related the brain to collar dose, achieving dose distributions in the brain similar to ours. Garzón et al^42^ measured brain doses by positioning a Rando phantom opposite to the operator during actual clinical interventional procedures (ie, at the patient’s left side instead of the right). Consequently, the doses were higher in the right hemisphere rather than the left, showing a similar difference in the near and far side of the brain as in the current study (approximately a factor of two higher in the near side). Smeulders et al^34^ used Monte Carlo simulations to estimate brain dose from interventional cardiological procedures, resulting in up to a factor of 2.8 higher doses in the left side compared to the right.^34^

Roguin et al^17^^,^^18^ reported a disproportionate number of left-sided (compared to right-sided) brain tumours of different types, known to be associated with exposure to ionising radiation among interventional cardiologists and radiologists, suggesting this was due to occupational exposure. Indeed, our study, as well as other published papers,^17^^,^^29–32^^,^^34^^,^^43^^,^^44^ confirm that the left side of interventional cardiologist’s head is more exposed to scattered radiation than the right side. However, the authors of the aforementioned studies^17^^,^^18^ emphasised the limitation of their findings, interpreting observations from a “cluster” of cases for which the true distribution in the total population is not known. Even though no conclusions can be drawn from these findings, it still raises interesting questions regarding risk calculation when dose is heterogeneously distributed within an organ, which practically always is the case in both diagnostic and occupational exposure situations. Risk calculation following exposure to ionising radiation is per definition carried out after averaging the dose over the entire organ assuming homogenous dose distributions and equal radiosensitivity within the organ. This has been briefly investigated by Samei et al,^45^ dividing a simplified model of an organ into voxels and calculating the risk of malignant transformation in each voxel based on depth-dose distribution from different modalities and dose-response models from the BEIR VII-report.^46^ Depending on discontinuity in the dose distribution and difference in radiosensitivity within an organ it is suggested that mean organ dose could both over- and underestimate the cancer risk. However, the paper only attempts a first exploration of the effect and therefore the implications for the current study are unclear.

For eye lens dose, the CCs estimated in this paper are lower compared to previously published papers^29^^,^^30^ but within the same order of magnitude. This has multiple possible explanations, such as differences between the studies with regards to x-ray spectra, operator position relative to the patient and x-ray equipment, or operator height, as the eye lens dose has been shown to decrease as operator height increases.^47^ In addition, there remain uncertainties due to TLD and *P*_KA_-meter-calibration although the contributions of these factors are relatively small (see Methods).

The absorbed dose to the left eye lens compared to the brain for the beam projections investigated in our study, is higher by a factor three to seven, in concordance with results by Fetterly et al^33^ and Moriña et al.^48^ Thus, a rough approximate of the dose to the brain in interventional cardiologists can be obtained from the more easily estimated eye lens dose.

**Figure 7.**
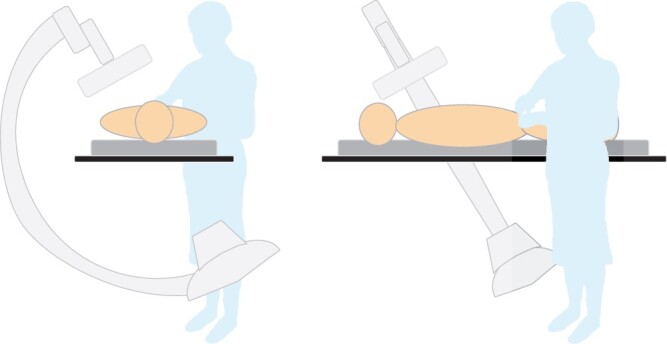
Illustration of operator position relative to the c-arm for LAO (left panel), and CRAN (right panel) projections when using right femoral access. Note that the operator is closer to the entrance site of the photons on the patient, compared to opposite projections such as RAO and CAUD, resulting in more scatter to the operator.

The results also show that the performing cardiologist is subject to more scatter in LAO and CRAN projections. This is because back- and side scattered photons must travel a shorter path before exiting the patient relative to forward scattered photons, when using right femoral access (Figure 7). Furthermore, the absorbed dose to the brain and eye lens per unit *P*_KA_ increases with tube voltage and added total filtration. The explanation for this is probably twofold. First, the fraction of Compton scattering, relative to photoelectric absorption is larger, following the increase in energy of the spectrum; second, higher energy photons will travel a larger distance into the head before attenuated. The increase in dose from scattered radiation has previously been reported in several studies. Ferrari et al^32^ demonstrated monotonically increasing dose to all parts of the brain per unit *P*_KA_ for mean x-ray beam energies in the range of 40-70 keV. Perisinakis et al^49^ measured ambient dose equivalent for multiple projections, room positions and beams energies demonstrating the same trend. As an example, in the LAO30-projection, at a position similar to the one in this work, an increase in dose equivalent at eye level of approximately 1.5 was observed when changing tube voltage and filtration from 70 kV, 8 mm Al to 81 kV, 11 mm Al. Sanchez et al^50^ measured *H*_p_(10) at the c-arm in interventional cardiac procedures, and reported up to factor of 2.6 higher dose equivalent unit per *P*_KA_ going from no added filtration to 1 mm Al + 0.4 mm Cu.

Increasing patient size did not increase scatter to the operator per unit *P*_KA_, as previously shown by Vano et al^30^; as a matter of fact, a slight decrease was noted for the evaluated projection (LAO30_XL; Table 3). This could be due to increased attenuation of scattered radiation due to the longer path a photon must traverse before exiting the patient after its initial scatter event. Note, however, that the total *P*_KA_ per procedure is likely to increase when treating larger patients, as the ADRC must compensate for the increase in attenuation between the x-ray tube and FPD. Consequently, the total amount of scatter, that is, the dose to the operator, is likely to increase when treating larger patients.

Using clinical data, taking the distribution of *P*_KA_, mean *P*_KA_ per procedure, and clinical workload into account, the mean dose per procedure or year can be calculated. For example, in the authors’ hospital, the annual absorbed dose to the whole brain of the interventional cardiologist, using the weighted CC from Table 3, is estimated to 8 mGy. The calculation is based on routine clinical workload where an operator typically performs 150 coronary angiographies (CA), 10 percutaneous coronary interventions (PCIs), 150 combined CA/PCI procedures and 150 transcatheter aortic valve implantation (TAVI) procedures. Mean *P_KA_* -values per procedure is for CA: 10 Gycm^2^, for PCI: 25 Gy cm^2^, for combined CA/PCI: 29 Gy cm^2^ and for TAVI 33 Gy cm^2^.^51^^,^^52^ The dose to the brain corresponds to a 1/5th of that of a CT-brain examination.^53^ Moreover, the absorbed dose to the left side of the brain is 10 mGy (using weighted CC), and for worst-case geometry (LAO30FL) the dose is doubled. According to recent literature, the absorbed dose threshold for circulation disease in the brain is 0.5 Gy.^54^ This practically implies that an interventional cardiologist in our hospital would exceed the dose threshold after 64 years of working life if a ceiling mounted lead protective shield is not used. While such a long career is highly unlikely, it must be noted that higher workloads, *P*_KA_-values per procedure or an excessive use of unfavourable geometries could substantially reduce the time needed to exceed these thresholds; for example, the suggested European Diagnostic Reference Levels (DRL: s) for interventional cardiac procedures^55^ are higher than the *P_KA_*-values stated above by a factor of three to four. Furthermore, if heterogeneity in the dose distributions indeed play a role, that is, if only part of the brain need exceed the threshold to induce circulation disease at some location, even less time may be needed. Finally, the CCs in this work are calculated assuming the operator use femoral access. Several studies have shown that radial access could increase the radiation dose to the interventional cardiologist by up to a factor of two.^31^^,^^40^^,^^56^^,^^57^ For eye lens dose, the absorbed dose threshold of 0.5 Gy for induction of cataract would be exceeded after 13 years working life (using the weighted CC) or 8 years (worst-case geometry) in our hospital.

It must be noted that these evaluations are conservatively estimated, without the use of radiation protection devices normally used in the everyday clinical practice. If a ceiling suspended lead protective articulated screen is used correctly, the scatter dose to the operator can be reduced by at least 75%.^40^^,^^58–60^ Additional shielding as leaded eyewear could reduce the dose to the eye lens by at least another 30%.^40^^,^^61^^,^^62^ However, it must be emphasised, the necessity to always consult local expertise, that is, medical physics experts (MPE) in the clinic, on the use of radiation protection tools instead of merely adopting what the industry suggests. The MPE follow recent literature and can suggest the best approach according to the latest scientific findings. One example from the last decade, is the use of a radioprotective cap claiming to reduce the dose to the brain; while closer investigations have shown its effectiveness to be highly dependent on the circumstances of use.^61^^,^^63^^,^^64^

### Limitations

It must be noted that the study described in this work does suffer from some inherent limitations, one of which being that operators are unlikely to hold their heads in the exact same spot during an entire procedure. Even though the operator position assumed can be thought of as an average while standing next to the patient table, an operator with proper training and awareness of radiation protection, could, for example, step back during image acquisition if possible. Furthermore, the cumbersome nature of TLD measurements entailed that only a limited number of measurements were performed in this study. More data including smaller steps in rotation/angulation, radial access geometries, more beam qualities and variation in operator height would certainly make estimation of organ doses more accurate. An additional limitation is the lack of the abdomen of the patient phantom. Like some other papers^29^^,^^33^ we have used a thorax phantom to simulate the patient. This decision likely results in overestimation of scatter for some projections where the abdomen would partially shield the operator from radiation, the weighted CC presented in this work therefore being a likely upper bound.

## Conclusion

Conversion coefficients in terms of dose to water per unit air-kerma area product in interventional cardiologists’ brain and eye lenses under actual scatter conditions have been estimated. These were calculated for a range of standard irradiation projections in interventional cardiological procedures and using femoral access. Results show higher doses in the left side of the cardiologist’s brain and to the left eye lens. This work provides the clinic with a monitoring tool for dose estimation to the cardiologist’s brain and eye lenses. Using these CCs, clinical workload and clinical exposure data, a coarse estimate of brain and eye lens dose can be made in any clinic.
